# Safety and efficacy of resistive polymer versus forced air warming in total joint surgery

**DOI:** 10.1186/s13037-017-0126-0

**Published:** 2017-04-14

**Authors:** Melanie F. Sandoval, Paul D. Mongan, Michael R. Dayton, Craig A. Hogan

**Affiliations:** 1 Department of Orthopedics, Division of Adult Reconstruction, University of Colorado-Denver, School of Medicine, 12605 E 16th Avenue, Aurora, CO 80045 USA; 2 Department of Anesthesiology, University of Colorado-Denver, School of Medicine, 12401 E. 17th Avenue, Mail Stop B113 7th Floor, Aurora, CO 80045 USA; 3 University of Colorado Hospital, Anschutz Medical Campus, 12605 E. 16th Avenue, Aurora, CO 80045 USA

**Keywords:** Forced-air warming, Polymer resistive warming, Surgical site infection, Orthopedic patient safety

## Abstract

**Background:**

Forced-air warming is used as a mechanism to prevent hypothermia and adverse outcomes associated with hypothermia among patients undergoing surgery. Patient safety in healthcare includes the use of devices and technology that minimize potential adverse events to patients. The present study sought to compare the capabilities of patient warming between two different devices that use different mechanisms of warming: forced-air warming and non-air warming.

**Methods:**

One hundred twenty patients undergoing total hip or total knee arthroplasty received patient warming via a forced warming device or non-air warming fabric conductive material. The project was part of a quality improvement initiative to identify warming devices effective in maintaining normothermic patient core temperatures during orthopedic surgery.

**Results:**

Forced-air warming and non-air warming achieved similar results in maintaining the core temperature of patients undergoing total knee or hip arthroplasty. No adverse events were reported in either group. Operating room staff observed that the non-air warming device was less noisy and appreciated the disposable covers that could be changed after each surgical case.

**Conclusions:**

These findings demonstrate that hypothermia is achieved by both forced-air and non-forced air warming devices among total knee and hip arthroplasty patients. The potential for airflow disruption is present with the forced-air warming device and does not exist with the non-forced air device. The disruption of laminar airflow may be associated with surgical site infections. The disposable covers used to protect the device and patient have potential implications for surgical site infection. Quality improvement efforts aimed to enhance patient safety should include the implementation of healthcare equipment with the least known or suspected risk.

## Background

The temperature in the operating room environment combined with the use of general anesthesia agents place surgical patients at an increased risk for the development of perioperative hypothermia. Perioperative hypothermia is associated with a number of adverse outcomes, including: surgical site infection [1–3] morbid cardiac events [4]; increased blood loss and transfusion requirements [5]; increased length of hospital stay [6]; adrenergic activation; thermal discomfort; and, decreased drug metabolism [7–9]. Improving patient safety using a health system approach includes the prevention of error and adverse outcomes through continuous quality improvement, including the application and use of the best healthcare engineering devices available [10]. Active warming devices are patient safety devices used to prevent hypothermia and the adverse outcomes associated with perioperative hypothermia.

Methods to warm surgical patients are routine practices used to prevent hypothermia and the associated poor patient outcomes. Devices used to warm the patient include forced-air warming devices, conductive-fabric warmers, and water-circulating warmers. Forced air warming is a popular method used to warm patients prior to the induction of anesthesia, intraoperatively, and post-operatively. A continued debate as to which warming device (specifically, resistive-polymer conductive fabric warming with the Hot Dog® and forced-air warming with the Bair Hugger®) is superior in preventing perioperative hypothermia remains a topic of controversy. The potential for the Bair Hugger® to increase the risk of surgical site infection has also been debated. Avidan and colleagues conducted a study in which agar plates were placed in the air stream of a forced-air warming device, concluding that potentially pathogenic microbes were present in the air stream [11]. The findings from this study suggest an association between the presence of microbes in the air stream (specifically, the hose) of forced-air warming devices and the potential for increased surgical site infection. However, the actual occurrence of surgical site infection among patients warmed using a forced-air warming device were not evaluated. The polymer fiber resistive warming device does not use air flow, which eliminates the possibility of disruption of the laminar air flow; requires disposable covers; and, operates without noise. The present project was not designed to determine the incidence of infections.

The literature is inconclusive in identifying the most efficient and effective warming device for surgical patients. Studies have concluded that forced air warming devices and resistive polymer air-free warming devices are equivalent in preventing perioperative hypothermia. In a randomized-control trial conducted by Brandt and colleagues [6], forced air warming and conductive fabric warmers were equally effective in maintenance of core temperatures, mean body temperatures, and mean skin temperatures among surgical patients. Similary, Kimberger et al. [12] compared the efficacy of the Bair Hugger® (Arizant, Eden Prairie, MN) to the polymer fiber resistive warming device (Hotdog®, Augustine Biomedical, Eden Prairie, MN) and found that metabolic heat production, cutaneous heat loss, and core temperature capabilities were equally effective among a group of non-anesthetized healthy volunteers. The Kimberger [12] study did not include patients who had received general anesthesia, which may or may not have influenced the findings.

McGovern and colleagues investigated the capacities of patient warming devices to disrupt laminar air flow and found that patients who had received forced-air warming had significantly greater rates of deep joint infection (3.8, *p* = 0.024) compared to patients who had received warming with an air-free fabric warming device [13]. A major limitation of McGovern study [13] included inconsistent prophylactic antibiotic regimens among patients during the study period. In contrast, a literature review conducted by Kellam et al., concluded that forced air-warming devices are not associated with increased risk of SSI and that forced-air warming devices were preferable over alternative methods of perioperative patient warming [14].

In light of the potential for increased infection rates [13], and subsequent legal ramifications associated with forced-air warming devices, an anesthesiologist, orthopedic surgeon, and team of nurses, and operating room staff compared the standard forced-air warming device, the Bair Hugger® with a resistive-polymer fabric-warming device, the Hot Dog®. The Bair Hugger® uses forced-air to maintain normothermia. The forced air mechanism of warming has been shown to increase particulates over warming areas in simulation studies [14], which, in theory, may translate to forced-air and the subsequent disruption of airflow increasing the potentiation of microbes in the sterile surgical field. Active warming using a resistive polymer is achieved by the electrical flow of current transmitted through a reusable resistive polymer blanket covered by a disposable polypropylene sheet. None of the patients in the current quality improvement project developed infections linked to either warming device.

The following quality improvement project expands upon the study conducted by Brandt et al. [6]. As part of a continuous effort to improve patient safety and quality, advances in healthcare tools and technology are periodically evaluated. The primary goal of this project was to compare the safety and effectiveness in patient warming capabilities between the standard of care warming device (forced-air warming) and the Hot Dog® resistive polymer warming device. The secondary goal was to explore the cost of the Bair Hugger® and the cost of the Hot Dog® to our institution. The findings of this project confirm the findings of previous studies, concluding that forced-air warming and resistive-polymer fabric warming are equally effective in preventing hypothermia among patients undergoing orthopedic surgery.

The aim of this report is to confirm previous studies’ findings that forced-air warming devices and resistive-polymer fabric warming devices do not differ in capability of patient warming. Further, we aim to increase awareness of alternative patient warming devices among health care professionals concerned with microorganisms that may be found in the airflow produced by the Bair Hugger® [11] and the potential for wound proliferation with these microorganisms, documenting that the resistive-polymer fabric device is an equally effective alternative, in terms of maintaining normothermia during surgery. This project explores the safety, efficacy, and capability of warming between a standard, forced-air warming device used at an academic institution and a thermal warming device.

## Method of improvement

The affiliated institution approved the project as quality improvement. A total of 120 (*N* = 120; *n* = 60 (50%), *n* = 60(50%)) patients undergoing total hip or knee arthroplasty were included in the quality improvement project. Augustine Medical Device Company provided HotDog® Warming Devices and supplies for up to 60 patients for evaluation.

Sixty patients were actively warmed with the Bair Hugger® (standard of care), forced-air warming device, and immediately after induction with anesthesia. Based on chronological order by date and time of the scheduled surgery, the sixty patients were warmed using the Hot Dog® (alternative warming) resistive polymer device. Patients were not randomized and no power analysis was conducted, based on the intent of the project. The method of warming received by the patient was solely based on order of chronology. Specifically, the first sixty patients scheduled received forced-air warming with the standard of care (Bair Hugger®) and the second group of patients scheduled received warming with the Hot Dog®. Patient core temperature was used as the primary measurement.

The attending anesthesiologist documented core temperatures obtained via an indwelling Foley catheter every fifteen minutes during the entirety of the surgery. Core temperatures were also documented at the initial phase of perioperative care, including: patient entry into the preoperative bay; arrival to the operating room; immediately before transfer from the operating room to the post-anesthesia care unit (PACU); and, upon admission to and discharge from PACU.

Demographics included: Age, weight (kg), body mass index (BMI), type of operation (total knee or total hip arthroplasty), anesthesia administered (spinal or general), estimated blood loss, and American Society of Anesthesiology Score (ASA Class). Estimated blood loss was determined by the visual estimate of blood loss based on saturation of dry sponges (for example, 4x4 sponge-10 ml; Ray-techs-10-20 ml; and lap sponges-50-100 ml) and the volume of blood in the suction canister at the end of the operation, after closing and after dressing was applied to the incision.

Descriptive and frequency analysis were also performed for OR time (minutes) the patient arrived in the operating room to the time the patient was transferred to PACU (“wheels in to wheels out”); cut to close time (minutes), with “close time” defined as the time the dressing was fully applied; and, core temperatures. An F-test was performed demonstrating equal variance between the two populations. In order to test differences in warming patients, a two-sample *t*-test assuming equal variance was conducted on the lowest core temperatures between warming devices.

## Findings

Mean skin temperature preoperatively, intraoperatively, and postoperatively offered similar intraoperative temperature maintenance between patients warmed with the forced-air warming device (Bair Hugger®) versus the patients warmed with a resistive-polymer warming device (Hot Dog®). The *t*-test did not reach statistical significance, *t* (118), 1.704, *p* > 0.05.

Patients in both groups remained free of injury, including burns, and surgical site infections. Hospital surveillance data (90 days post-op) revealed zero SSIs among patients warmed with either device. Complications relative to the devices did not occur. Demographic characteristics of the patients in each group were similar, as were ASA Class, estimated blood loss (defined as estimated volume of any blood loss on the surgical field, drapes, sponges, floor, and in the suction canister), operation type, and OR time (Table 1).

**Table 1:** Patient demographics

Characteristic	Bair Hugger (*n* = 60) Mean ± Standard Deviation *n* = frequency, % = percentage		Hot Dog (*n* = 60) Mean ± Standard Deviation *n* = frequency, % = percentage	
Age (years)	60.2 ± 11.4		63.9 ± 12.7	
Weight (kg)	84.5 ± 25.8		79.5 ± 18.7	
Body Mass Index	29.5 ± 7.8		28.2 ± 6.0	
Operation (hip, knee)	*n* = 32(53%), *n* = 28(47%)		*n* = 32(53%), *n* = 28(47%)	
Anesthesia (spinal, general)	*n* = 26(43.33%), *n* = 34(56.67%)		*n* = 36(60%), *n* = 24(40%)	
OR Time (“wheels in to wheels out”) (minutes)	171.97 ± 50.72		147.07 ± 32.70	
^a^Cut to Close Time	113.7 ± 45.70		87.98 ± 29.04	
^b^Estimated Blood Loss (milliliters)	294 ± 253		244 ± 236	
American Society of Anesthesiology Score (ASA Class)	Class	*n* = frequency (% = percentage)	Class	*n* = frequency (% = percentage)
	1	*n* = 3 (5%)	1	*n* = 1(1.67%)
	2	*n* = 24(40%)	2	*n* = 19(31.67%)
	3	*n* = 30(50%)	3	*n* = 38(63.33%)
	4	*n* = 3(5%)	4	*n* = 2(3.33%)

^a^“Close time” defined as the time at which the dressing has been fully applied

^b^Estimated blood loss is a visual estimate of blood loss based on the saturation of dry sponges and the volume (ml) collected in the suction canister(s) at the end of the surgery, after the incision is closed and the dressing is fully applied

The core temperature remained similar between both groups at the initial core temperature, preoperative temperature, lowest intraoperative core temperature, final intraoperative core temperature, and post-anesthesia care unit (PACU) core temperature (Table 2). Bair Hugger® cut to close times (*M =* 113.7, *SD =* 45.69) were not similar to HotDog® cut to close times (*M =* 87.98, *SD =* 29.04). The average OR time (minutes), defined as patient entry into the operating room until patient transfer to PACU (“wheels in to wheels out”) was not similar among individuals warmed using the Bair Hugger® (*M =* 171. 97, *SD =* 50.72) compared to patients warmed with the HotDog® (*M =* 147.07, *SD =* 32.70).

**Table 2:** Core temperatures and time from last Operating room (OR) temperature to Post-Anesthesia Care Unit (PACU)

Perioperative Phase (°C)	Bair Hugger (*n* = 60) Mean ± Standard Deviation	Hot Dog (*n* = 60) Mean ± Standard Deviation
Operating room (OR) Temperature	19.3 ± 1.0	19.5 ± 1.1
Preoperative temperature	36.5 ± 0.3	36.6 ± 0.4
Initial core temperature	36.0 ± 0.4	36.1 ± 0.5
Lowest core temperature	35.6 ± 0.5	35.7 ± 0.5
Final core temperature in OR	35.6 ± 0.5	35.8 ± 0.5
PACU temperature	36.3 ± 0.3	36.3 ± 0.3
Time from last OR to PACU Temp (minutes)	15.0 ± 6.8	16.4 ± 7.2

**Fig. 1:**
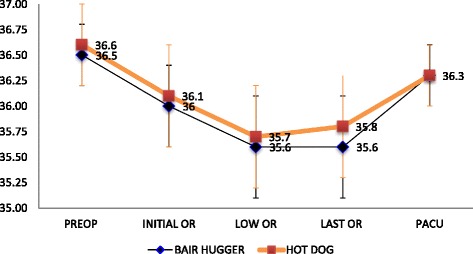
Mean core temperatures (ºC) among patients warmed with Bair Hugger® or Hot Dog®. Mean core temperatures (ºC) of patients during the perioperative phase of care from preop to PACU. Patient core temperatures did not differ during any point of care (preop, initial OR temperature, lowest OR temperature, last OR temperature, PACU) between the HotDog® (*n*=60) and Bair Hugger® warming devices. Notes: Preop refers to preoperative phase; initial OR refers to the first temperature taken prior to anesthetic induction; low OR refers to the lowest core temperature recorded intraoperatively; last OR refers to the last temperature recorded before transfer to post-anesthesia care unit (PACU); PACU refers to the first temperature taken after admission to PACU from the OR

After induction of anesthesia, initial core temperature changes in the Bair Hugger® Group and Hot Dog® Group were 36.0 °C ± 0.4 °C and 36.1 °C ± 0.5 °C, respectively; these values remained similar. The lowest intraoperative core temperatures was similar as well between patients warmed using the Bair Hugger® (35.6 °C ± 0.5 °C) and patients warmed using the Hot Dog® (35.7 °C ± 0.5 °C). The final intraoperative core temperatures between patients warmed using the Bair Hugger® (35.6 °C ± 0.5 °C) and patients warmed using the Hot Dog® (35.8 °C ± 0.5 °C) and PACU temperature between the Bair Hugger® (36.3 °C ± 0.3) and Hot Dog® (36.3 °C ± 0.3) also remained similar (Fig. 1). Core temperature changes did were similar throughout the intraoperative and postoperative phases of care.

## Discussion

In the context of an era in which patient safety, including mechanisms used to promote safety and avoid adverse outcomes, the application of devices with the least-associated risk should be fully explored. Although conflicting evidence relative to patient warming device exists, surgical patients should receive the best care and be treated with the best devices, even with suspected risk. Further, Federal litigations against the company associated with forced-air warming devices elevates the need to explore alternative means of patient warming. The current project found equal patient warming capabilities between forced-air warming and polymer-resistive fabric warming.

Patients in both groups maintained similar core temperatures and no adverse events occurred relative to the warming devices in either group. Advantages of the polymer-resistive fabric warming includes less noise and a mechanism of non-air warming that does not impede the purpose of the laminar air flow—to promote clean air and decrease the likelihood of contamination of the surgical site. Warming device pricing differs, with forced-air warming devices being less costly.

Further research should be conducted to investigate warming devices and surgical site infections vs. warming devices and particulate counts. Particulate counts may not represent actual risk of surgical site infections. Healthcare analyses of cost relative to savings are also lacking and may be useful to individuals or institutions when choosing a warming device. Provider, staff, and patient preference may be the determinant of devices selected for patient warming, as the capability of each warming device was found to be equally effective in preventing normothermia among orthopedic patients undergoing total arthroplasty at our academic-affiliated institution during the course of the described quality improvement project.

## Conclusion

In conclusion, the Bair Hugger® and Hot Dog® devices were similar in preventing hypothermia among patients undergoing orthopedic surgery. No adverse outcomes occurred in either group because of the warming device. The Hot Dog® warming device is noise free and does not disrupt the laminar airflow. Cost and usability may be the greatest factors in organizational decisions to select the product used to warm surgical patients.

## Abbreviations

C: Degrees Celsius
EHR: Electronic health record
OR: Operating room
PACU: post-anesthesia care unit
